# Supplementary material for: Kojic Acid Gene Clusters and the Transcriptional Activation Mechanism of Aspergillus flavus KojR on Expression of Clustered Genes
Source: J Fungi (Basel). 2023 Feb 15;9(2):259. doi: 10.3390/jof9020259 (PMC9961346; doi:10.3390/jof9020259)
Supplement: Supplementary file 1 [file jof-09-00259-s001.zip › jof-2106709-supplementary.pdf]

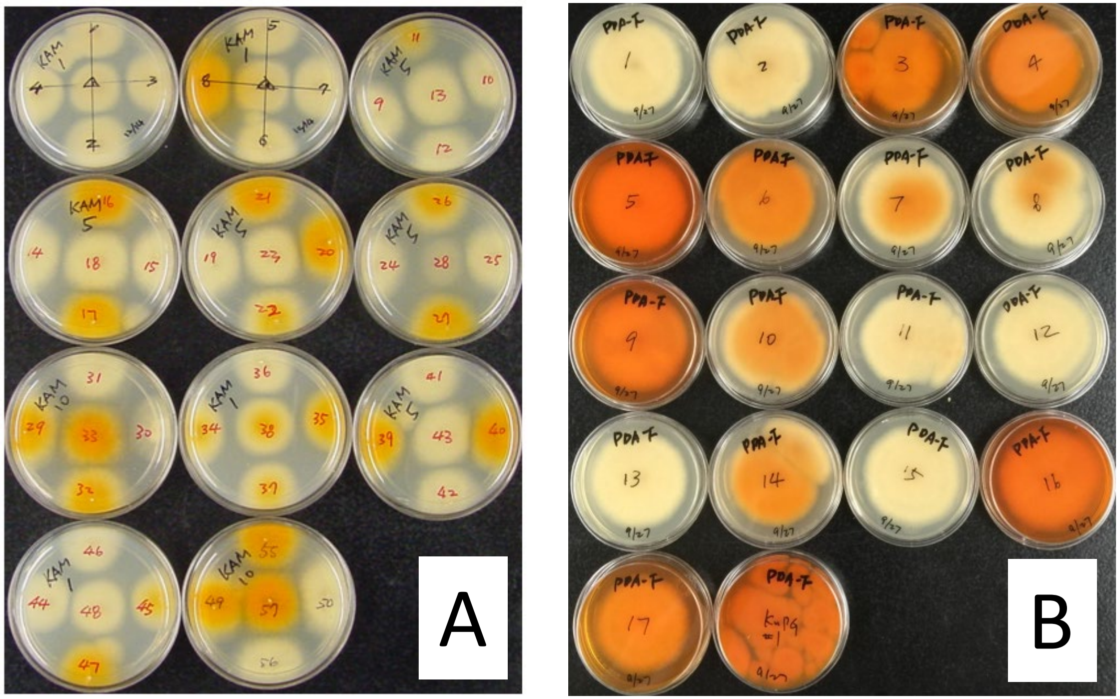

Figure S1. Examination of KA production by *kojR* complemented transformants of *A. flavus* ΔkojR#4. (A) *kojR* expression was driven by *A. nidulans gpdA* promoter, and transformants were transferred onto KAM plates. (B) *kojR* expression was driven by *A. flavus gpiA* promoter, and transformants were transferred onto PDA plates supplemented with ferric ion. Color intensity is indicative of the KA amount produced.

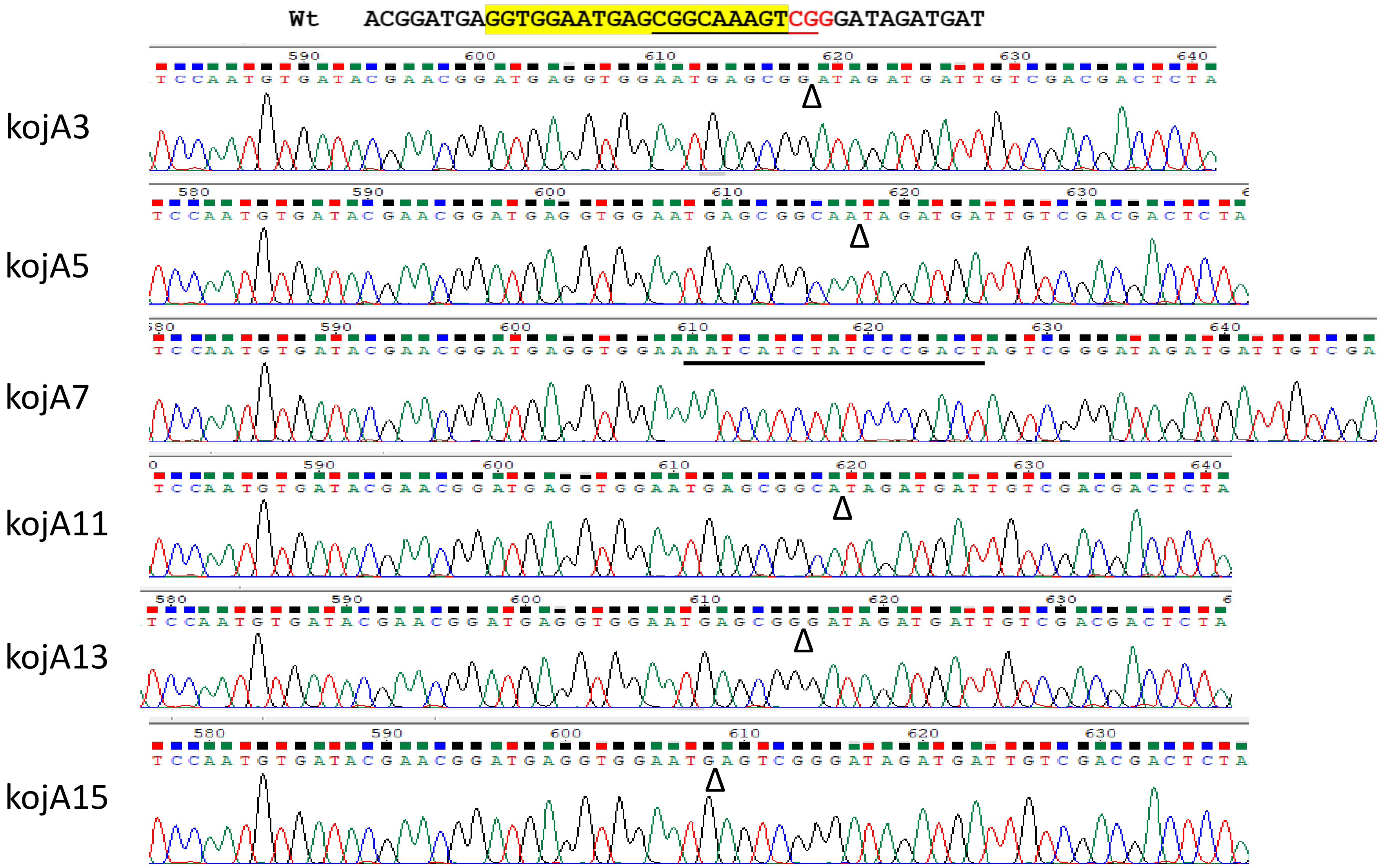

Figure S2. Sequencing chromatograms showing locations of deletions and an insertion in the KojR-binding motif of *A. flavus kojA* promoter.

```
kojT3   431     GCTCGTATGCGTCTACTTATAGTATTAGTCGTTGAACCTAGTACCTAATAATTCTGCTTG  490
                ||||||||||||||||||||||||||||||||||||||||||||||||||||||||||||
Sbjct   159806  GCTCGTATGCGTCTACTTATAGTATTAGTCGTTGAACCTAGTACCTAATAATTCTGCTTG  159747

Query   491     AATTATATCATAGACTGACATACCTCATGTAAGCCCTTCCTCCTCCTTCGGCGTCGAATA  550
                ||||||||||||||||||||||||||||||||||||||||||||||||||||||||||||
kojT3   159746  AATTATATCATAGACTGACATACCTCATGTAAGCCCTTCCTCCTCCTTCGGCGTCGAATA  159687

Query   551     AGGATTTTCGAAGCCATTCAGCGGCT  576
                ||||||||||||||||||||||||||
Sbjct   159686  AGGATTTTCGAAGCCATTCAGCGGCT  159661
```

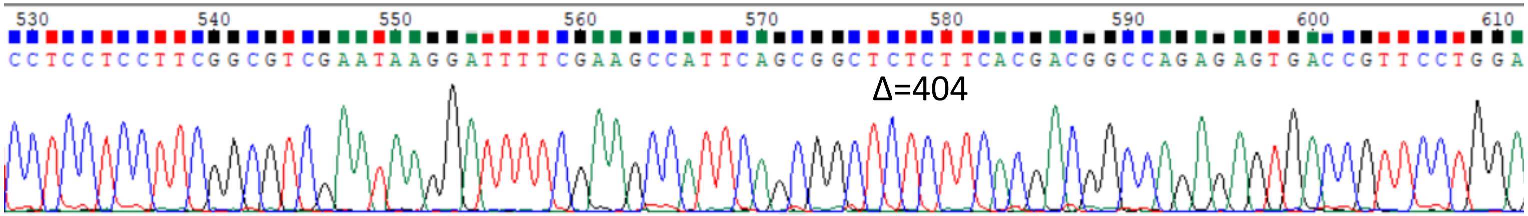

Wt   ATTTTCGAAGCCATTCAGCGGCTAAGTCGGGCATGACGGA

```
kojT3   578     TCTTCACGACGGCCAGAGAGTGACCGTTCCTGGAGTCAATCTCCGCCGCGCCTCCGAAAT  637
                ||||||||||||||||||||||||||||||||||||||||||||||||||||||||||||
Sbjct   159257  TCTTCACGACGGCCAGAGAGTGACCGTTCCTGGAGTCAATCTCCGCCGCGCCTCCGAAAT  159198

kojT3   638     CTGTGAGCGCGTCAATACTAAAACACTTTTCATTGTCGGGTTCGATGGCCCGGACGACCA  697
                ||||||||||||||||||||||||||||||||||||||||||||||||||||||||||||
Sbjct   159197  CTGTGAGCGCGTCAATACTAAAACACTTTTCATTGTCGGGTTCGATGGCCCGGACGACCA  159138

kojT3   698     GCTCAATCCCAAAAACTGGTCTATAGGGCGGAAATGGGCGACATTGGGCATTGTAGGCAC  757
                ||||||||||||||||||||||||||||||||||||||||||||||||||||||||||||
Sbjct   159137  GCTCAATCCCAAAAACTGGTCTATAGGGCGGAAATGGGCGACATTGGGCATTGTAGGCAC  159078
```

159661-159257=404 deleted in kojT3

Figure S3. Sequencing chromatogram showing the breakpoint and sequence alignment for identifying a large deletion that disrupted the suggested motif in the *kojT* promoter and extended to the *kojT*-coding region in a KA-producing *A. flavus* mutant.

```
kojT18   76     CACCAACTGCCCCCATGCTTCATGGATGGCCAGTTGTTCCACCATGCACATCATAGAGGC  135
                ||||||||||||||||||||||||||||||||||||||||||||||||||||||||||||
CA14   160795   CACCAACTGCCCCCATGCTTCATGGATGGCCAGTTGTTCCACCATGCACATCATAGAGGC  160736

kojT18   136    CATCGGGGCTCACCAGGATCCGGAACTGGTGTCTTTGGTCTATTCTGACACCGCAGACGT  195
                ||||||||||||||||||||||||||||||||||||||||||||||||||||||||||||
CA14   160735   CATCGGGGCTCACCAGGATCCGGAACTGGTGTCTTTGGTCTATTCTGACACCGCAGACGT  160676

kojT18   196    TAGCGTAAATGATGAG  211
                ||||||||||||||||
CA14   160675   TAGCGTAAATGATGAG  160660
```

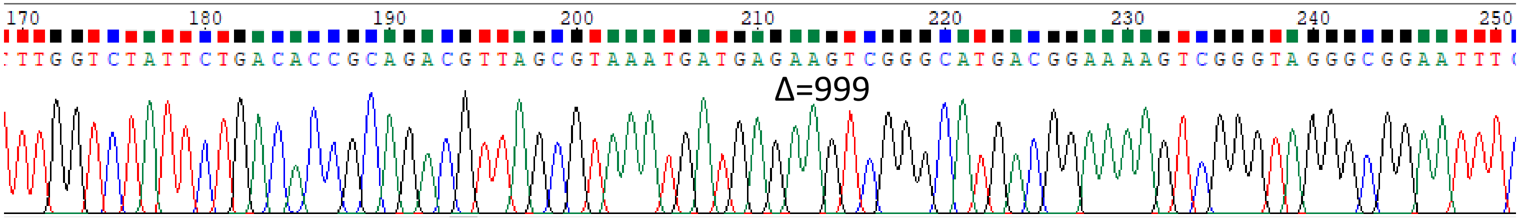

Wt   ATTTTCGAAGCCATTCAGCGGCTAAGTCGGGCATGACGGA

```
kojT18   212    AAGTCGGGCATGACGGAAAAGTCGGGTAGGGCGGAATTTCCGTTGACGCAATGGAAGCAC  271
                ||||||||||||||||||||||||||||||||||||||||||||||||||||||||||||
CA14   159660   AAGTCGGGCATGACGGAAAAGTCGGGTAGGGCGGAATTTCCGTTGACGCAATGGAAGCAC  159601

kojT18   272    CACTAGAAATAAATACTCTGAAAGAGCACATCTATCCCAGCCGAGGAATAAAAACACTTG  331
                ||||||||||||||||||||||||||||||||||||||||||||||||||||||||||||
CA14   159600   CACTAGAAATAAATACTCTGAAAGAGCACATCTATCCCAGCCGAGGAATAAAAACACTTG  159541

kojT18   332    ACAGATGCTGCTCAATTAATTGCCTCCCTCCCAATTCAttttttttATTCTGTGGCGTAT  391
                ||||||||||||||||||||||||||||||||||||||||||||||||||||||||||||
CA14   159540   ACAGATGCTGCTCAATTAATTGCCTCCCTCCCAATTCATTTTTTTTATTCTGTGGCGTAT  159481
```

160660-159660-1=999 deleted in kojT18

Figure S4. Sequencing chromatogram showing the breakpoint and sequence alignment for identifying a large deletion that disrupted the suggested motif in the *kojT* promoter and extended to the upstream *kojR*-coding region in an *A. flavus* mutant that was unable to produce KA.

Table S1. Primers used in vector construction, qRT-PCR, and sequencing

Primer name	Sequence (5’→3’)	Use
5kojR-Sac	CGAGTTGCTTTAGACCGAGG	Disruption
5kojR-B	CTAATTCGGGATCCCGTTATCC	Disruption
3kojR-Xho	AATCTCG AGAGTCAAAGACGGTTATTC	Disruption
3kojR-Sp	GTTGAACCTTGTTCGGTCAGC	Disruption
kojR-OE	ACTATAGCGGCCGCATGTCGTTGAATACCGACGATTCC	Expression, PCR
kojR-STOP	TCCTGCATTATCTATATCTC	Expression
GPI-H	ATAAAGCTTCGCACTGTACGTAGTAGTA	Promoter swapping
GPI-Not	TATAGTGCGGCCGCTGTTATGTGATTTCTTCTAATGGAGA	Promoter swapping
kojA -qF	CCGTATCATCCACACCGAGG	qRT-PCR
kojA -qR	AACCGGAAGAGCATCTGCAA	qRT-PCR
kojR-qF	AATACCGACGATTCCGGTCG	qRT-PCR, copy number
kojR-qR	TTTCCTCTTGCGCAGTTTGC	qRT-PCR, copy number
kojT -qF	GGCCAAAGCACGCATATCAG	qRT-PCR
kojT -qR	CGAACAGGAAAATAGCGCG	qRT-PCR
18S-F	TTCCTAGCGAGCCCAACCT	qRT-PCR
18S-R	CCCGCCGAAGCAACTAAG	qRT-PCR
U6-F-P	ATACTGCAGTTCTCTTTAGAATTCAACTGTGGGT	CRISPR
U6-R-K	TATGGTACCACATATTTAAAAAAAGTCTCCTGCC	CRISPR
kojA_F	GGTGGAATGAGCGGCAAAGTGTTTTAGAGCTAGAAATAGCAAGTTAA	CRISPR
kojA_R	ACTTTGCCGCTCATTCCACCCTTGTTCTTCTTTACAATGATTTATATACC	CRISPR
kojT_F	AAGCCATTCAGCGGCTAAGTGTTTTAGAGCTAGAAATAGCAAGTTAA	CRISPR
kojT_R	ACTTAGCCGCTGAATGGCTTCTTGTTCTTCTTTACAATGATTTATATACC	CRISPR
kojA-FCk	GATCAACCGCAGTACACTCA	PCR, Sequencing
kojA-CkR	TCGTATCGCAAGCAGTAAGT	PCR, Sequencing
kojT-FCk	CGCTCGCCTTGGTGGCATGT	PCR, Sequencing
kojT-CkR	TCACAGTGGAGTCGATGGAG	PCR, Sequencing
kojR1500	TGCCAGATGGCATGATCCTG	PCR, Sequencing
kojR1650	GATCTCAAAGACTACTCTCCT	PCR, Sequencing

Table S2. Sequences of the *kojA* and *kojT* promoter regions used for the identification of KojR DNA-binding motif by MEME

A_aflatoxiformans_kojA	TATGAAGAGGCAGGTAGTTATAGTCTAGAGATGGTTATGTTCGGTTCAAGAATCTTGAGAAGAATGATTGTTGAAGTGTAGAGGCATACCC<br>CCAAAGGATCTCCATCGCTTTATATCCTTCCCTTCAGCAAGATAGCTTGACTCACTCCCATGGGCATGTCAAAAATGGTCCTTCCCTGATT<br>GTAACCCAACCTTGCCGGATAACGACACATGCACCCTGAAGCCAATTGCATCCAATGTGATACGAACGGATGAGGTGGAATGAGCGGCAAA<br>GTCGGGATAGATGATTGTCGACGACTCTAGAAGATCGTAACATACTGAATGAGCGTTTAATGCATGTCAGCGCTTGATAATGTCACATCCG<br>CGGCGATG
A_alliaceus_kojA	TTTGGAGAGACAAGTTATTGTAGTTCAAAGATGATATGTGTTCAGTTAAAGGATTAGAGGACTGGTTTCTGAAGAGAAGAGGACCAATGCA<br>GAAGAGGTCCTTCGACTTATATCCTTTCCTCAGTGTCGTATCCTGACTTACGGTGGTGGGTCGTGTCAAGGATGTTCTTCTCATCATTGTA<br>ACCTAAACATTTGTTGATAGTGATGTATGCATCCTGAAATCGGCCGTGTCCAATACGAGAAGAACGGATGAGGTGGAATGAGCGGCAAAGT<br>CGGGAAAGATGATTGTCGACAACTATAGATGAGGGTTAAAACACCTCTCGAGCGTCTTGTGCATGTCAGCACTTGATAATGTCACATCCAC<br>GGTGATTC
A_arachidicola_kojA	TATGAAGAGGGAGGTAGTTATAGTCTAGAGATGATTATGTTCAGTTCAAGAATCTTGAGAAGAATGGTTGTTGAAATGTAGAGGGATACTC<br>CCCAAGGATTTCCATCCCTTTATATCCTTCCCTTCAGCAAGACAGCTTGACTCACTCCCATGGGCGTGTCAAGGATGCTCTTTTCCTGATT<br>GTAACCCAACCTTGCCGGATAACGACACATGCACCCTGAAGCCAATTGCATCCAATGTGATACGAACGGATGAGGTGGAATGAGCGGCAAA<br>GTCGGGATAGATGATTGTCGACGACTCTAGAAGACCGTAACATACTGAATGAGCTTTTAATGCATGTCAGCGCTTGATAATGTCACATCCG<br>CGGCGATG
A_bertholletius_kojA	GATGAAGAGACAAGTAGTTTTAGATGAGAGATGAGTGTGTTCGGTTCAAGAATTTTGAGAAGGCTGGCTCGTGAAGTGTAGAAGAATAGCC<br>CCCACGGCGTCCCCATCGCTTTATATCCTTCTATCCGCCGTGATTGTTTGAGTCACTCCTGTGGGCATATCAAGGATGCTCAATCCCTGAT<br>TGTAACGCAACATTCCCAAACCATGACACATGCACCGCGAAACCAATCGCATCCAACGCGATACGAACGGATGAGGTGGAATGAGCGGCAA<br>AGTCGGGATAGATGATTGTCGGCGACTGTAGAGGATGGCAACATACTGAATGAGCTCTTAATGCATGTCAGCGCTTGATAATGTCACATCT<br>GCGACGACG
A_burnettii_kojA	TTTGGAGAGACAAGTTATTGTAGTTCAGAGACGATATGTGTTCAGTTGAAGGATTAGAGGACTGGTTTCTGAAGAGAAGAGGACCAATGCA<br>GAAGAGGTCCTTCGACTTATATCCTTTTCCTCAGTGTCGTATCCTGACTTACGGTGGTGGGTCGTGTCAAGGATGTTCTTCTCATCATTGT<br>AACCTAAACATTCGTTGATAGTGATGTATGCATCCTGAAATCGGCCGTGTCCAATACGAGAAGAACGGATGAGGTGGAATGAGCGGCAAAG<br>TCGGGAAAGATGATTGTCGACAACTATAGATGAGGGTTAAAACACCTCTCGAGCGTCTTGTGCATGTCAGCACTTGATAATGTCACATCCA<br>CGGTGATT
A_caelatus_kojA	ATGAATAGGCAGGTAGTTATAGTCTAGAGATGATTATGTTCGGTTCAAGAGTCTTGAGAAGAATGGTTGTTGAAGTGTAGAGGAATAGCCC<br>CCCAAGGATTCCCGTCGCTTTATATCCTTCCCTTCAGCAGGATAGCTTGAGTCACTCCTATGAGCATGTCAAGGATTCTCCTTCCCTGATT<br>GTAACCCAACACACCCGGATAACGACACATGCACTCTGAAGCCAATCGCATCCAATGTGATACGAACGGATGAGGTGGAATGAGCGGCAAA<br>GTCGGGATAGATGATTGTCGACGACTCTAGAAGATCGCAACATACTCACTGAGCTTTTAATGCATGTCAGCGATTGATAATGTCACATCCG<br>CGGTGAT
A_coremiiformis_kojA	GTCAAAGTGAATTACGAAGTAGTTCTAATCTGGAGTTGATTCGGTTCCGTTCAAGAAGCTCGCAAGAAGTGGAAGTTGACGAAAAGAGAAA<br>CAGTTCGGAAGAGGTTCATCCCCTTATATTGCTTTCCTCCGTGTCGTATCTGGACTCACTCTGGTGGGCCATAGCTAGGATTTTCTTCTCC<br>TGATTGTAACCCAAGATTCCAAGATAGCGACGGATGCATGCTGACACCAATGGCAACCAAGGCAATACGAACGGGTCAGGTGGAATGAGCG<br>GCAAAGTCGGGACCGATGATTGTCGACGACTATAGATGATTCTCAAATGCTTGAGTTGTACTGCTAACAATG
A_flavus_kojA	TATGAAGAGGCAGGTAGTTATAGTCTAGAGATGGTTATGTTCGGTTCAAGAATCTTGAGAAGAATGATTGTTGAAGTGTAGAGGCATACCC<br>CCAAAGGATCTCCATCGCTTTATATCCTTCCCTTCAGCAAGATAGCTTGACTCACTCCCATGGGCATGTCAAAAATGGTCCTTCCCTGATT<br>GTAACCCAACCTTGCCGGATAACGACACATGCACCCTGAAGCCAATTGCATCCAATGTGATACGAACGGATGAGGTGGAATGAGCGGCAAA<br>GTCGGGATAGATGATTGTCGACGACTCTAGAAGATCGTAACATACTGAATGAGCGTTTAATGCATGTCAGCGCTTGATAATGTCACATCCG<br>CGGCGAT
A_hancockii_kojA	TTTGAAGAGACAAGTAATAGTGAATATTATAGTGATTGCCGTAGTCGTCTATAGATAACAATCAATTCTTAGGAAACTTGAAGAGGCTGGT<br>GGTTTGAAAATGAGAAGCGCCCCTAGGGACTCAATCGCTTTATATCCTTTTCTTCAGCGTCGTATCTTGACTCCCCACGGTGGATCATGTC<br>AAGGGTCTCCCTCCCATGATTGCAACCCAACATTCGAAGATAGCGACATACGCATCCTGAAACGAATCGCATCCAATGCGATACGAACGGA<br>AGAGGTGGAATGAGCGGCAAAGTCGGGATAGACCATCTTCGATGACAAGAGAAGATCATTAAACAGCTGTTGAGTTCCTCATGCATGTCAG<br>CTTTTGATAATGTC

A_leporis_kojA	CTTGAAGAAACAAGTAGTAGTAAAAGTGGTAGTCGTAGTAGTCCAGAGGTGATACTCTAAGAAGCTCAACGAGGCTGGTGTTTGAAGAAAT<br>GAGAAGCTTCCCACCAAGGACTCGTTGACCTTATATCCCTTTCTTCAGTGTCGTAGCTTGACTCTCCTCGATGGGACATGTCAAGGATCTT<br>CTTCCTATGATTGCAACCTAACATCCGAATATAGCGACATACACATCCTAAAACTAGCAGTATCCAATGCGAGACGAACGGAAGAGGTGGA<br>ATGAGCGGCAAAGTCGGGATGGAGCGTTTTCGATGATAATGGAAGATCATAAAGCAGGTGTTGAGCTCCTCATGCGTGTCAGCTTTTGATA<br>ATGTCACATC
A_luteovirescens_kojA	TATGAAGAGGCGGGTAGTTATAGTCTAGAGATGATTGTGTTTGGTTCTAAAGCTTGAGAAAACTGGTTGTTGAAGTAGGTAGGAATAGCCC<br>CCAAGGCTTCCCATAGCTTTATATCCTTCTCGCCAGCAGGGTAGCTTGACTCACTTCCGTGGCCATGTCAAGGGTGCTCTTTCCATGATTG<br>TAACCCAACATTCCCCGATAGCGACACATGCACTCTGAAGCCAATCGCATTCAATGTGATACGAACGGATGAGGTGGAATGAGCGGCAAAG<br>TCGGGATAGATGATTGTCGACGACTCCAGAAGATCGTAACATACTTGAATGAGTTTTTGATGCATGTCAGCGCTTGATAATGTCACATCCG<br>CGGCGAT
A_minisclerotigenes_kojA	TATGAAGAGGCAGGTAGTTATAGTCTAGAGATGGTTATGTTCGGTTCAAGAATCTTGAGAAGAATGATTGTTGAAGTGTAGAGGCATACCC<br>CCCAAGGATCTCCATCGCTTTATATCCTTCCCTTCAGCAAGATAGCTTGACTCACTCCCATGGGCATGTCAAGGATGGTCCTTCCCTGATT<br>GTAACCCAACCTTTGCCGGATAACGACACATGCACCCTGAAGCCAATTGCATCCAATGTGATACGAACGGATGAGGTGGAATGAGCGGCAA<br>AGTCGGGATAGATGATTGTCGACGACTCTAGAAGATCGTAACATACTGAATGAGCGTTTAATGCATGTCAGCGCTTGATAATGTCACATCC<br>GCGGCGAT
A_nomiae_kojA	TATGAAGAGGCAGGTAGTTATAGTCTAAAGATGATTCTGTTCGGTTCAAAAGACTTGAGAAAGGTGGTTGTTGAAGTAGGGAGGAATAGCC<br>CCCAAGGCTTCACATCGCTTTATATCCTTCCCCCAGCAGGATGGCTTGACTCACTCCGGTGGCCATGTCAAGGGTGCTCTTTCCCTGATTG<br>TAACCCAACATTCCCAGATAACGACATATGCATCCTGAAGCCAATCGCATCCAATGTGATACGAACGGATGAGGTGGAATGAGCGGCAAAG<br>TCGGGATAGATGATTGTCGACGACTCCAGAAGATCGTAACATACTTGAATGAGGTTTTGATGCATGTCAGCGCTCGATAATGTCACATCCG<br>CGGCGA
A_novoparasiticus	TATGAAGAGGCAGGTAGTTATAGTCTAGAGATGATTATGTTCGGTTCAAGAATCTTGAGAAGAATGGTTGTTGAAATGTAGAGGCATACTC<br>CCCAAGGATTTCCATCCCTTTATATCCTTCCCTTCAGCAAGACAGCTTGACTCACTGCCATGGGCATGCCAAGGATGCTCTTTTCCCTGAT<br>TGTAACCCAACCTTGCCGGATAACGACACATGCAACCTGAAGCCAATTGCATCCAATGTGATACCAACGGATGAGGTGGAATGAGCGGCAA<br>AGTCGGGATAGATCACTGTCGACGACTCTAGAAGATTGTAACATACTGAATGAGCTTTTAATGCATGTCAGCGCTTGATAATGTCACATCC<br>GCGGCGATG
A_oryzae_kojA	TATGAAGAGGCAGGTAGTTATAGTCTAGAGATGGTTATGTTCGGTTCAAGAATCTTGAGAAGAATGATTGTTGAAGTGTAGAGGCATACCC<br>CCAAAGGATCTCCATCGCTTTATATCCTTCCCTTCAGCAAGATAGCTTGACTCACTCCCATGGGCATGTCAAAAATGGTCCTTCCCTGATT<br>GTAACCCAACCTTGCCGGATAACGACACATGCACCCTGAAGCCAATTGCATCCAATGTGATACGAACGGATGAGGTGGAATGAGCGGCAAA<br>GTCGGGATAGATGATTGTCGACGACTCTAGAAGATCGTAACATACTGAATGAGCGTTTAATGCATGTCAGCGCTTGATAATGTCACATCCG<br>CGGCGAT
A_parasiticus_kojA	TATGAAGAGGCAGGTAGTTATAGTCTAGAGATGATTATGTTCGGTTCAAGAATCCTGAGAAGAATGGTTGTTGAAATGTAGAGGCATACTC<br>CCCAAGGATTTCCATCCCTTTATATCCTTCCCTTCAGCAAGTCAGCTTGACTCACTCCCATGGGCATGTCAAGGATGCTCTTTTCCTGATT<br>GTAACCCAACCTTGCCGGATAACGACACATGCACCCTGAAGCCAATTGCATCCAATGTGATACGAACGGATGAGGTGGAATGAGCGGCAAA<br>GTCGGGATAGATGATTGTCGACGACTCTAGAAGATTGTAACATACTGAATGAGCTTTTAATGCATGTCAGCGCTTGATAATGTCACATCCG<br>CGGCGAT
A_pseudocaelatus_kojA	TATGAATAGGCAGGTAGTTATAGTCTAGAGATGATTATGTTCGGTTCAAGAGTCTTGAGAAGAATGGTTGTTGAAGTGTAGAGGAATAGCC<br>CCCCAAGGATTCCCGTCGCTTTATATCCTTCCCTTCAGCAGGATAGCTTGAGTCACTCCTATGAGCATGTCAAGGATTCTCCTTCCCTGAT<br>TGTAACCCAACATACCCGGATAACGACACATGCACTCTGAAGCCAATCGCATCCAATGTGATACGAACGGATGAGGTGGAATGAGCGGCAA<br>AGTCGGGATAGATGATTGTCGACGACTCTAGAAGATCGCAACATACTCACTGAGCTTTTAATGCATGTCAGCGATTGATAATGTCACATCC<br>GCGGTGAT
A_pseudonomius_kojA	TTTGAAGAGGCAGGTAGTTATAGTCTGAAGATGATTCTGTTCGGTTCAAAAGACTTGAGAAAGGTGGTTGTTGAAGTAGGGAGGAATAGCC<br>CCCAAGGCTTCCCATCGCTTTATATCCTTCTCCGCAGCAGGATAGCTTGACTCACTCCGGTGGCCATGTCAAGGGTGCTCTTTCCCTGATT<br>GTAACCCAACATTCCCGGATAACGACATATGCACCCTGAAGCCAATCGCATCCAATGTGATACGAACGGATGAGGTGGAATGAGCGGCAAA<br>GTCGGGATAGATGATTGTCGACGACTCCAGAAGATCGTAACATACTTGGATGAGCTTCTGATGCATGTCAGCGCTTGATAATGTCACATCC<br>GCGGCG

A_pseudotamarii_kojA	TATGAATAGACAGGTAGTTATAGTTTAGAGATGATTATGTTCGGTTCAGGAATCTTGAGAAGAATGGTTATTGAAGTGTAGAGGAATAGCC<br>CCCCAAGGATTCCCGTCCCTTTATATCCTTCCCTTCAGCAGGATGGTTTGAGTCACTCCCATGAGCATGTCAAGGATTCTCTTTCCCTGAT<br>TGTAACCCAACATATCCGGATAACGACACATGCACTCTGAAGCCAATCGCATCCAATGTGATATGAACGGATGAGGTGGAATGAGCGGCAA<br>AGTCGGGATAGATGATGTTCGACGACTCTAGAAGATCGTAACATACTCACTGAGCTTTTAATGCATGTCAGCGATTGATAATGTCACATCC<br>GCGGTGA
A_sergii_kojA	TATGAAGAGGCAGGTAGTTATAGTCTAGAGATGATTATGTTCGGTTCAAGAATCTTGAGAAGAATGGTTGTTGAAATGTAGAAGCATACTC<br>CCCAAGGATTTCCATCTCTTTATATCCTTCCCTCCAGCAAGGCAGCTTGACTCACTCCCATGGGCATGTCAAGGATGCTCTTTTCCTGATT<br>GTAACCCAACCTTGCCGGATAACGACACATGCACCCTGAAGCCAATTGCATCCAATGTGATACGAACGGATGAGGTGGAATGAGCGGCAAA<br>GTCGGGATGGATGATTGTCGACGACTCTAGAAGATTGTAACATACTGGATGAGCTTTTAATGCATGTCAGCGATTGATAATGTCACATCCG<br>CGGCGAT
A_sojae_kojA	TATGAAGAGGCAGGTAGTTATAGTCTAGAGATGATTATGTTCGGTTCAAGAATCCTGAGAAGAATGGTTGTTGAAATGTAGAGGCATACTC<br>CCCAAGGATTTCCATCCCTTTATATCCTTCCCTTCAGCAAGTCAGCTTGACTCACTCCCATGGGCATGTCAAGGATGCTCTTTTCCTGATT<br>GTAACCCAACCTTGCCGGATAACGACACATGCACCCTGAAGCCAATTGCATCCAATGTGATACGAACGGATGAGGTGGAATGAGCGGCAAA<br>GTCGGGATAGATGATTGTCGACGACTCTAGAAGATTGTAACATACTGAATGAGCTTTTAATGCATGTCAGCGCTTGATAATGTCACATCCG<br>CGGCGATG
A_tamarii_kojA	TATGAATAGGCAGGTAGTTATAATCTAGAGATTATTATATTTTGTTCAAGAATCTTGAAAAGAATGGTTGTTGAAGTCTAGAGGAATAGCC<br>CCCCAAGGATTCCCGGCGCTTTATATCCTTCCCTTTAGCAGGATAGTTTGAGTCACTCCCATGAGCATGTCTAGGATTCTCTTTCCCTGAT<br>TGTAACCCAACATATCCGGATAACGACACCTGCACTCTGAAGCCAATCGCATCCAATGTGATACGAACGGATGAGGTGGAATGAGCGGCAA<br>AGTCGGGATAGATGATTGTCGACGACTCTAGAAGATCGCAACATACTCACTGAGCTTTTAATGCATGTCAGCGATTGATAATGTCACATCC<br>GCGGTGAT
A_transmontanensis_kojA	TATGAAGAGGCAGGTAGTTATAGTCTAGAGATGATTATGTTCGGTTCAAGAATCTTGAGAAGAATGGTTGTTGAAATGTAGAGGCATACTC<br>CCCAAGGATTTCCATCCCTTTATATCCTTCCCTTCAGCAAGACAGCTTGACTCACTCCCATGGGCATGTCAAGGATGCTCTTTTCCTGATT<br>GTAACCCAACCTTGCCGGATAACGACACATGCACCCTGAAGCCAATTGCATCCAATGTGATACGAACGGATGAGGTGGAATGAGCGGCAAA<br>GTCGGGATAGATGATTGTCGACGACTCTAGAAGATTGTAACATACTGAATGAGGTTTTAATGCATGTCAGCGCTTGATAATGTCACATCCG<br>CGGCGAT
A_alliaceus_kojT	TACAGAAATGTCAAGACTGATTGTATGATGGAACAGTGTCCGCACGCATCTACTTATAGTGTTAGACAAATATTGTAATACCTAATTATTT<br>CTACTTAAATTACACTATGAGCTGGGATAACATCTGTATGTTCTTTGACTTTCCCTGGTTCCGAATGAGAAATTTCGAAGCCATTCAGCGG<br>CTATGTCGGACGCGACGGGAAAGTCGGATAGGGCGGAATTCGGGGTTGACGCAACGACAGCACCACTGGAAATAAATACTCCCGGAAAGAA<br>TAACTATTCTAGCCAGTGAAAGAGAACATTCAGACAAATGTTGCTCGAATAATTGTGTCTCTCCAAGAAAACACTTCCTCGTTCTGCTCAG<br>TTTCTCCGCGCTTCGCAATT
A_arachidicola_kojT	TGCAGGAAATTGAGGATTATTTACACGATCAGAAAGTGCTAGTATGCGTCTACTTATAGTATTAGTCAATGAACCTGGTGCCTAATAATTC<br>AAGGAAAATTCTATCATAGACTCACATACCTCTGTAAGCCCTTCCTCGTCCTTCGGCGCCGAATAAGGATTTTCGAAGCCACTCGGCGGCT<br>AAGTCGGGCATGACGGAAAAGTCGGGTAGGGCGGAATTCCCGTTGACGCAATGGAAGCACCGCTAGAAATAAATACTCTGAAGAGAGCACA<br>TTTGTTCTAGGCGAGGAGTAAAAACATTTGACAGATGCTGCTCAGTTAATTGCCTTCCTTCCAATTTCATATTTATTCTGCGGCGTATGTC<br>TTGAACGCCTCGCAACG
A_bertholletius_kojT	TGTAGGAAATGAGAACTATCTACAGGGCTCAAAAGTGCTTGCCTGCGTCTTCTTATAGTCTTAGTCAATGGACCTAACACCCCATAAATCT<br>GCATAGATTCGACTATAGACTAGCATAGCTCTGGTACACTCTTCCTCGTCCTTAGGTTCGAGCTGAATAAGGATAGTCGAAGCCATTCAGC<br>GGCTAAGTCGGGCATGACGGAAAGGTCGGTTAGGGCGGAATTATGGATTGACGCAATAGAAGCAGCATGAGAAATAAATACGCTGAAACGA<br>GCTCAATGATTCGAGCTGAGGAGTAACCACATCTACCAGATGTTGCTTAATTGATATTTTGTCTTCCGTTTCCTATTTTCCTTTTCACTGT<br>CCGTAGTGTGTGTCTTGAGCGTCTCAGAACG
A_burnettii_kojT	TACAGAAATGTCAAGACTGATTGTATGATCGAACAGTGTCCGCACGCATCTACTTATAGTGTTAGACAAATATTGTAATACCTAATTATTT<br>CTACTTAAATTACACTATGAGCTGGGATAACATCTGTATGTTCTTTGACTTTCCCTGGTTCCGAATGAGAAATTTCGAAGCCATTCAGCGG<br>CTATGTCGGACGCGACGGGAAAGTCGGATAGGGCGGAATTCGGGGTTGACGCAACGACAGCACCACTGGAAATAAATACTCCCGGAAAGAA<br>TAACTATTCTAGCCAGTGGAAGAGAACATTCAGACAAATGTTGCTCGAATAATTGTGTCTCTCCAAGAAAACACTTCCTCGTTCTGCTCAG<br>TTTCTCCGCGCTTCGCAATT

A_caelatus_kojT	TGCAGAAAATTGAGGACTAATTACACGATCAGAAAGTGCTCGTATGCGTCTACTTATAGTATTAATCGATGAGCCTAGTACCTATTAATTC<br>TGCGTCCATTATATCATAGACTGACGTACCTCTTGTAAGCTCTTTCTCGCCCTTTGGCGCCGAATAAGGATTTTCGAAGCCATTCAGCGGC<br>TAAGTCGGGCATGACGGGAAAGTCGGTTAGGGCGGATTTCGGGTTGACGCAATGGAAGGAACACTAGAAATAAATACTCCGAAAAGAGCAC<br>ATTTATTCTTGCCGAGGAGTAAAAACATTTGACAGATGTTGTTTTAGTTTAATATCTCTCTCCCAATTCCAATCTTTATTTTGTGGTGTGT<br>GTCGTGAGCGCCCCGCAACG
A_coremiiformis_kojT	AAGCATGTCCGTGTGGCTCTACTTATAGTATTAGTCCATGACTGTAGTGACTGATAATCTCTACATTGGTTACACTACAGATTGGCATTCT<br>TTTGCTTTGGTGTTGCGTTATTCGGTTCCAAACAAGAAATTTCCATGCCATTTGGCGGGTAAGTCGGGCATGACGGGTAGGGCGGGTAGGG<br>CGGACTGCGGGTTTGATGCACCGCTGGAAATAAATACTCCGAAAAGATCAATTAATCCAGTCAGTGTGTGAGAATATTGAGACATATGCTG<br>ATCGATTGATTGCCTCCTTCCCAGCTCTTTTCTTTTCCTTTCTTTTTTTGTTTGTGTGTGTGTGTGCCTGGAACACCTGGCAACA
A_flavus_kojT	TGCAGGAAATTGAGGACTATTTACACGATCAGAAAGTGCTCGTATGCGTCTACTTATAGTATTAGTCGTTGAACCTAGTACCTAATAATTC<br>TGCTTGAATTATATCATAGACTGACATACCTCATGTAAGCCCTTCCTCCTCCTTCGGCGTCGAATAAGGATTTTCGAAGCCATTCAGCGGC<br>TAAGTCGGGCATGACGGAAAAGTCGGGTAGGGCGGAATTTCCGTTGACGCAATGGAAGCACCACTAGAAATAAATACTCTGAAAGAGCACA<br>TCTATCCCAGCCGAGGAATAAAAACACTTGACAGATGCTGCTCAATTAATTGCCTCCCTCCCAATTCATTTTTTTTATTCTGTGGCGTATG<br>TCTTGAACGCCTCGCAACG
A_hancockii_kojT	TACCAGTTAAGAGGATTCAGGAGTGTCTGTATGCGTCTACTTATAGTCTTATACAATCAATGCAGTAAATAATCATATCTACCTTGGTTAC<br>ACTATAGACTGGCATTTATTCCGTATGTTCTTTTTCCTTCTCTGGTTCCGAACGAGAATTTTCGAAGTCATCCAGCGGTTAAGTCGGACGC<br>AGCGGAAAAGTCGGGTAGGGCGGAATCCCGCTTTGACGCAATAGAAGCACCCACGAGTGGAAATAAATACTCCTGTAATTTGCAAATATTG<br>CACACAGTGAGTAAGGACCCCAAACAGATGTTGCTCCATTGATCCGGTCTGTTCTGTTTCTTGTTTTCTTTCGCAATCGCGGTCGCGATAC<br>AACC
A_leporis_kojT	TACAGGAATGATGGCTACTGAAGAGTGTCCGTATGTGTCTACTTATAGTACTAGGCAATGAATGTAGTGGGTAAGCAATTCTACTTGGGTT<br>ACACTATAGACTGGCATGACTTTGTATGTTGTTTTTCCTCTAGTTCCGAACAAGAAATTTTCGATGTCATTCAACGGCTAAGTCGGACACA<br>GCGGAAAAGTCGGGTAAGGCGGAATTCAGCTTTGACGCAATAGTAGCACGAGTGGAAATAAATACTCCGGCGATTGCAATTATTGCACACG<br>GTGAGTAAAGATCGCAGACAAACATTGTTGCTCAATTGATAGTGGCTGTCATTTCTATAAATTTTTTTTTTTCCTTTTTCCTTCCCTATAT<br>TTGGTCGCGGTACAAACC
A_luteovirescens_kojT	TGCAGGAAATGAGGACTATTTACACCACCAGGAAGTGCTCGTATGCGTCTACTTATAGTATTAGTTAATGGACCTAGGACTTAATAAATCT<br>GTTTGGATTATATCATAGACAGACATACCTCTTGTAAACTGTTCCTTGCCCTTTGGCGCCGAATAAGGATTTTCGGAGCTATTCAGCGGTT<br>AAGTCGGGCATGACGGGAAAGTCGGTTAGGGCGGAATTCGGGTTGACGCAATGGAAGCACCACCAGAAATAAATACTCTGAAAGAGCACAT<br>TTGTTCTAGCCGAGGAGTAAAAACGTTTGACAGATGTTGCTTAATTGACTGCCTTTCTCCCAATTCCTTTTTGTATTTTCTGGGTTGTGTC<br>CTGATCGCCTCGCAACG
A_minisclerotigenes_kojT	TGCAGGAAATTGAGGACTATTTACACGATCAGAAAGTGCTCGTATGCGTCTACTTATAGCATTAGTCATTGAACCTAGTACCTAGTAATTC<br>TGCTTGAATTATATCATAGACTGACATACCTCTTGTAAGCCCTTCCTCCTCCTTCGGCGCCGAATAAGGATTTTCGAAGCCATTCAGCGGC<br>TAAGTCGGGCATGACGGAAAAGTCGGGTAGGGCGGAATTTCCGTTGACGCAATGGAAGCACCACTAGAAATAAATACTCTGAAAGAGCACA<br>TCTATCCTAGCCGAGGAATAGAAACATTTGACAGATGCTGTTCAATTAATTGCCTTCCTCCCAATTCATTTTTTTTATTCTGTGGCGTATG<br>TCTTGAACGCCTCGCAACG
A_nomiae_kojT	TGCAGGAAATGAGGACTATTTACAGAACCCGACAGTGCTCGTATGCGTCTACTTATAGTATTAGTTAATAGAGCTAGGACTTAATCATTCT<br>GTTTGGATTATATCATAGACGAACATACCTCTTCTAAGCTCCTCCTTGTTCTTTGGTGCCGAAAAACGGTTTTCGAAGCCATTCAGCGGCT<br>AAGTCGGGCATGACGGGAAAGTCGGTTAGGGCGGAGTTCGGGTTGACGCAATGGAAGCACCACTAGAAATAAATACTCTGAAAAGAGCACA<br>TTTAGTCTAGCCGAGGAGTGAAAACATTTGAGCAGATGTCGCTTGATTGATTCCTTTCTCCTAATTTCTTTTTTATTTTGTGGTGTGTGTC<br>TTGAGCGCCTCGCAACG
A_novoparasiticus_kojT	TGCAGGAAATTGAGGATTATTTACACGATCAGCAAGTGCTAGTATGCGTCTACTTATAGTATTAGTCAATGAACCTGGTACCTAATAATTC<br>AAGGAGAATTATATCATAGACTCACATACCTCTTGTAAACCCTTCCTCGTCCTTCGGCGCCGAATACGGATTTTCGAAGCCATTCAGCGGC<br>TAAGTCGGGCTTGACGGAAAAGTCGGGTAGGGCGGAATTCGCGTTGACGCAATGGAAGCACCACTAGAAATAAATACTCTGAAAAGAGCAC<br>ATTTATTCTAGCCGAGGAGTAAAACATTTGACAGATGCTGCTTAGTTAATTGCCTCCCTCCCAATTCTTTTTTTTATTCTGCGGCGTATGT<br>CTTGAACGCCTCGCAACG
A_oryzae_kojT	TGCAGGAAATTGAGGACTATTTACACGATCAGAAAGTGCTCGTATGCGTCTACTTATAGTATTAGTCGTTGAACCTAGTACCTAATAATTC<br>TGCTTGAATTATATCATAGACTGACATACCTCATGTAAGTCCTTCCTCCTCCTTCGGCGCCGAATAAGGATTTTCGAAGCCATTCAGCGGC

	TAAGTCGGGCATGACGGAAAAGTCGGGTAGGGCGGAATTTCCGTTGACGCAATGGAAGCACCACTAGAAATAAATACTCTGAAAGAGCACA<br>TCTATCCCAGCCGAGGAATAAAAACACTTGACAGATGCTGCTCAATTAATTGCCTCCCTCCCAATTCATTTTTTTTATTCTGTGGCGTATG<br>TCTTGAACGCCTCGCAACG
A_parasiticus_kojT	TGCAGGAAATTGAGGACTATTTACACGATCAGAAAGTGCTCTTATGGGTCTACTTATAGTATTAGTCAATGAACCTGGTACCTAATAATTC<br>AAAGAGAATCATAGCATAGACTCACATACCTCTTGTAAGCCCTCCCTCGTCCTTCAGCGCCGAATAAGGATTTTCGAAGCCATTCAGCGGC<br>TAAGTCGGGCATGACGGAAAAGTCGGGTAGGGCGGAATTCCCGTTGACGCAATGGAAGCACCATTAGAAATAAATACTCTGAAAAGAGCAC<br>ATTTATTCTAGCCGAGGAGTAAAACATTTGACAGATGCTGCTCAGTTAATTGCCTCCCTCCCAATTCCTTTTTTTATTCTGCGGCGTATGT<br>CTTGAACGCCTCGCAACG
A_pseudocaelatus_kojT	TGCAGAAAATTGAGGACTAATTACACGATCAGAAAGTGCTCGTATGCGTCTACTTATAGTATTAATCGATGAGCCTAGTACCTATTAATTC<br>TGCGTCCATTATATCATAGACTGACGTACCTCTTGTAAGCTCTTTCTCGCCCTTTGGCGCCGAATAAGGATTTTCGAAGCCATTCAGCGGC<br>TAAGTCGGGCATGACGGGAAAGTCGGTTAGGGCGGATTTCGGGTTGACGCAATGGAAGGAACACTAGAAATAAATACTCCGAAAAGAGCAC<br>ATTTATTCTTGCCGAGGAGTAAAAACATTTGACAGATGTTGTTTTGGTTGAATATCTCTCTCCCAATTCCAATTTTTATTTTGTGGTGTGT<br>GTCGTGAGCGCCCCGCAACG
A_pseudonomius_kojT	TGCAGGAAATGAGGACTATTTACAGAACCAGAAAGTGCTCGTATGCGTCTACTTATAGTATTAGTTAATAGACCTAGGATTTAATCATTCT<br>GTTTGGATTATATCATAGACGAACATATCTCTTCTAAGCTCTTCCTTGTCCTTTGGTGCCGAATAGCGGTTTTCGAAGCCATTCAGCGGCT<br>AAGTCGGGCATGACGGGAAAGTCGGTTAGGGCGGAGTTCGGGTTGACGCAATGGAAGCACCACTAGAAATAAATACTCTGAAAAGAGCACA<br>TTTAGTCTAGCCGAGTAGTGAAAACATTTGGCAGATGTCGCTTAATTGATTCCTTTCTCCCAATTTCTTTTTTTTTTATTTTGTGGTGTGT<br>GTCTTGAGCGCCTCGCAACG
A_pseudotamarii_kojT	TGCAGGAAATTGAGGACTATTTACACGATCAGAAAGGGCTCGTATGCGTCTACTTATAGTATTAGTCGATAGGCCTAGTACCTAGTAATTC<br>TGCTTGCATTATATCATAGACTGACGTACCTCTTGTAAGCTCTTTCTCGCCCTTTGGCGCTGAATAAGGATTTTCGAAGCCATTCAGCGGC<br>TAAGTCGGCCATGACGGGAAAGTCGGTTAAGGCGGATTTCGGGTTGACGCAATGGAAGGACCACTAGAAATAAATAGTCCGAAAAGAGCAC<br>ATTTATTCTTGCCGAGGAGTAAAAACATTTGACAGATGTTGCCTTAATTATCTCTCTCCCAATTCCTTTTATTATTTTGTGGTGTGTGTCT<br>TGAGCGCCTCGCAACG
A_sergii_kojT	TGCAGGAAATTGAGGATTATTTACACGATCAGAAAGTGCTAGTATGCGTCTACTTATAGTATTAGTCAATGAACCTGGTACCTAATAATTC<br>AAGGAGGATTATATCATACACTCACATACCTCTTGTAAGCCCTTCCTCGTCCTTCGGCGCCGAATAAGGATTTTCGAAGCCATTCAGCGGC<br>TAAGTCGGGCATGACGGAAAAGTCGGGTAGGGCGGAATTTTCGTTGACGCAATGGAAGCACCACTAGAAATAAATACTCTGAAAAGAGCAC<br>ATTTATTCTAGCCGAGGAGTAAAACATTTGACAGATGCTGCTCAGTTAATTGCCTCGCTCCCAATTCCTTTTTTTTATTCTGCGGCGTATG<br>TCTTGAACGCCTCGCAACG
A_sojae_kojT	TGCAGGAAATTGAGGACTATTTACACGATCAGAAAGTGCTCTTATGGGTCTACTTATAGTATTAGTCAATGAACCTGGTACCTAATAATTC<br>AAGGAGGATTATATCATAGACTCACATACCTCTTGTAAGCCCTTCCTCGTCCTTCAGCGCCGAATAAGGATTTTCGAAGCCATTCAGCGGC<br>TAAGTCGGGCATGACGGAAAAGTCGGGTAGGGCGGAATTCCCGTTGACGCAATGGAAGCACCATTAGAAATAAATACTCTGAAAAGAGCAC<br>ATTTATTCTAGCCGAGGAGTAAAACATTTGACAGATGCTGCTCAGTTAATTGCCTCCCTCCCAATTCCTTTTTCTATTCTGCGGCGCATGT<br>CCTGAACGCCTCGCAACG
A_tamarii_kojT	CACAGGAAATTGAGGACTATTTACACGATCAGAAAGTGCTCGTATGCGTCTACTTATAGTATTAATCCATGAGCCTAGTATCTAGTAATTC<br>TGCTTGCATTATATCATAGACTGACGTATCTCATGTAAGCTCTTTCTCGCCCTTTGGCGCCGAATAAGGATTTGCGAAGCCATTCAGCGGC<br>TAAGTCGGGCATGACGGGAAAGTCGGTTAGGGCGGATTCCGCGTTGACGCAATCGAAGCACCACTAGAAATAAATACTCCGAAAAGAGCAC<br>ATTTATTCTTGCCGAGGATTAAAGACATTTGACAAGTGTTGCTTTAATTGATTGTCTTTCTCCCAATTAACTTTTGTATTTTGTGGCGTGT<br>GTCTTGAGCGCCTCGCAACG
A_transmontanensis_kojT	TGCAGGAAATTGAGGATTATTTACACGATCAGAAAGTGCTAGTATGCGTCTACTTATAGTATTAGTCAATGAACCTGGTACCAAATAATTC<br>AAAGAGAATTATAGCATAGACTCACATACCTCTTGTAAGCCCTTCCTCGTCCTTCAGCGCCGAATAAGGATTTTCGAAGCCATTCAGCGGC<br>TAAGTCGGGCATGACGGAAAAGTCGGGTAGGGCGGAATTCCCGTTGACGCAATGGAAGCACCATTAGAAATAAATACTCTGAAAAGAGCAC<br>ATTTATTCTAGCCGAGGAGTAAAACATTTGACAGATGCTGCTCAGTTAATTGCCTCCCTCCTAATTCCTTTTTTTATTCTGCGGCGTATGT<br>CTTGAACGCCTCGCAACG

Table S3. Normalized expression levels of KA genes in *A. flavus* strains

Strain	Gene								
	kojA			*kojR*			*kojT*		
	48 h	72 h	Change[c]	48 h	72 h	Change	48 h	72 h	Change
KuPG[a]	4.16[b]	3.18	2.0	9.43	8.77	1.6	7.66	2.53	35.2
ΔkojR	7.32	8.09	0.6	25.38[d]	25.11[d]	1.2[d]	8.41	8.61	0.9
D-8	4.34	2.71	3.1	5.59	7.11	0.3	6.90	2.82	16.9
D-16	12.03	2.93	550.6	7.71	7.86	0.9	11.32	3.75	190.7
D-20	9.46	2.00	176.1	7.21	6.67	1.5	10.06	3.24	112.9
I-5	7.06	2.53	23.0	5.36	6.22	0.6	8.38	2.96	42.9
I-9	7.96	4.13	14.2	5.53	5.45	1.1	9.22	5.99	9.3
I-16	6.84	2.40	21.7	3.87	5.77	0.3	8.14	4.84	9.9

a: KuPG is the KA-producing control strain.

b: *A. flavus* 18S gene expression was used as the normalizer to obtain ΔCt values.

c: Fold of change in gene expression from 48 h to 72 h.

d: The Ct values are greater than 40, and the change thus is not meaningful.
